# Mechanically Active Supramolecular Systems

**DOI:** 10.1002/smsc.202300300

**Published:** 2024-03-19

**Authors:** Ke Shi, Xintao Lv, Jiawei Liu, Yiyang Lin, Jianwei Li

**Affiliations:** ^1^ State Key Laboratory of Chemical Resource Engineering Beijing Laboratory of Biomedical Materials Beijing University of Chemical Technology Beijing 100029 China; ^2^ MediCity Research Laboratory University of Turku Tykistökatu 6 20520 Turku Finland

**Keywords:** mechanical force, non‐equilibrium, self‐assembly, supramolecular materials

## Abstract

Mechanical sensing and transduction are integral to biological systems and have inspired research into the regulation of supramolecular self‐assembly via mechanical forces. This review presents an inaugural discussion on mechanically active supramolecular systems. It focuses on two primary mechanisms for modulating these systems: the incorporation of mechanophores and the application of mechanical forces to modulate non‐covalent interactions. Challenging the traditional view of mechanical forces as solely destructive, their constructive potential when harnessed through sophisticated design is showcased. Investigation is done on how external forces like ultrasound, stirring, vortex, tension, and compression can induce fluorescence in π‐conjugated systems, initiate hydrogelation, propel non‐equilibrium self‐assembly, and affect the structure of vesicles. The review also casts light on the prospective uses of mechanically active supramolecular systems in areas such as protein activation, drug delivery, and stress sensing, illustrating the nuanced role of mechanical forces as both disruptors and enablers in the creation of functional materials.

## Graphical abstract

This review summarizes recent advances in developing functional supramolecular systems that are responsive to mechanical stimuli such as compression, tension, stirring, shaking, ultrasound, and audible sound. Supramolecular systems are susceptible to mechanical stimuli at a low energy input, experiencing changes beyond the molecular scale, such as vesicle deformation, hydrogel formation/breakage, enzyme activation, chirality operation, and fibril alignment.

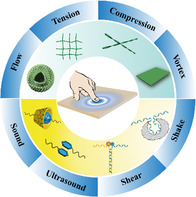

## 1 Introduction

Mechanical stimuli and sensing are fundamental in biology, underpinning numerous functions such as muscle contraction, touch, hearing, wound healing, and cell migration and division. Biological entities typically harness supramolecular transformations paired with secondary reporter systems to detect nuanced mechanical stresses. Cells, inherently mechanosensitive, are equipped to capture and transmute mechanical stimuli into biochemical cues. These cues, in turn, activate intracellular responses, including signalling pathways and transcriptional regulation.^[^ ^1^ ^]^ Red blood cells (RBCs), for instance, can readily alter their form in response to external forces and flow dynamics, facilitating their swift traversal through even the smallest blood vessels.^[^ ^2^ ^]^ An RBC's deformability is crucial when gauging its behaviour, and significantly impacts blood flow and viscosity. Endothelial cells display a similar capability; they perceive blood flow forces and interpret intricate shear stress conditions to steer both healthy and diseased responses.^[^ ^3^ ^]^ Likewise, mechanically active tissues, such as the myocardium, are attuned to mechanical inputs. An uptick in hemodynamic pressure, for example, boosts cardiomyocyte contractility.^[^ ^4^ ^]^ Our auditory system exemplifies the wonders of mechanical sensing. The ear captures ambient sound vibrations, converting them into nerve impulses. These impulses journey to the brain, where they are discerned as sounds.^[^ ^5^ ^]^

The indispensable role of mechano‐sensing in biology has propelled extensive research into mechanically active systems. Supramolecular materials—consisting of interacting modules that self‐assemble into distinct shapes and structures^[^ ^6^ ^]^—are adept at adjusting their physicochemical traits and configurations in response to external variations, such as changes in pH,^[^ ^7^ ^]^ temperature,^[^ ^8^ ^]^ enzyme action,^[^ ^9^ ^]^ light exposure,^[^ ^10^ ^]^ or electric fields.^[^ ^11^ ^]^ Their adaptability earmarks them as prime contenders for diverse applications, from smart materials to drug delivery.^[^ ^12^ ^]^ While polymers and organic materials’ mechanical chemistry is well‐chronicled,^[^ ^13^ ^]^ studies focusing on mechanically steered self‐assembled soft materials are fewer. Two probable explanations emerge: 1) some mechanical forces, like ultrasound, can disrupt self‐assembled constructs, and 2) transitioning a mechanical cue from a large to a minute scale in supramolecular systems lacks finesse.

In this review, we embark on an exploration of the myriad mechanical forces wielded to shape supramolecular systems. We catalogue both covalent (chemical) bonds and non‐covalent (physical) ties that are responsive to mechanical inputs. The spotlight is cast on the influence of mechanical forces on supramolecular π‐conjugated systems, their contribution to fostering self‐assembly and gelation, their role in vesicle deformation, and their part in initiating chirality by symmetry breaking and shaping non‐equilibrium systems.

## 2 Features of Various Mechanical Forces

**Figure 1:**
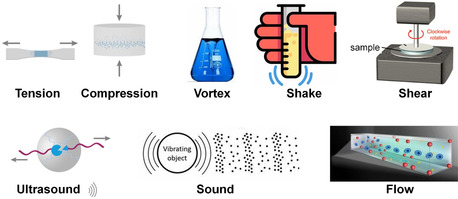
Mechanical forces applied to supramolecular systems.

Mechanical forces, inherent in both natural and man‐made environments, manifest distinct characteristics based on their unique characteristics and implications (**Figure** **1**). Compression, for instance, exerts a force perpendicular to a material's cross‐section, bringing its components closer together. In stark contrast to the compacting nature of compression, tension seeks to pull materials apart. It acts by elongating or stretching a material along its primary axis. The compressive and tensile forces can be readily applied to soft materials manually, or with the use of a load and dynamic mechanical analysis instruments that allow for quantitative and precise control of external forces. Compressive or tensile force‐responsive systems are mostly based on supramolecular materials such as films, hydrogels, and elastomers. Shear forces, operating parallel to a material's surface, introduce a different dynamic. They can cause layers or sections of a material to slide over one another. Shear forces can be generated to solution by vortex, shaking, stirring, or liquid flow under different conditions. Quantitatively, the flow or shear forces can be controlled with rheometer where the shear parameters of stress and strain rate are determined from measurements of torque and flow rate. This kind of force is intricately tied to fluid dynamics, especially in the context of blood flow.^[^ ^14^ ^]^ Consider, for example, the act of stirring a liquid in a container. The centre of the liquid experiences a tight, torsional flow, while the outer layers may swirl in a more spiral manner, highlighting the nuanced effects of shear. Ultrasound is defined as periodic vibration sound waves with a frequency above 20 kHz. The ultrasound radiation energy is known to generate thermal and non‐thermal effects. The non‐thermal effect is primarily associated with cavitation that can occur in native microbubbles or cavitation nuclei such as microbubbles or nanobubbles. The collapse of bubbles generates shear stress and shock‐waves, which impacts the structure of supramolecular systems. Sonication uses an ultrasonic bath or probe to apply sound energy to a solution containing soft materials. Traditional use of ultrasound at lower frequencies has primarily been for diagnostic imaging purposes, whereas the development of biocompatible high intensity focused ultrasound in the frequency range of 550 kHz–1.1 MHz has allowed this particular modality to expand into non‐invasive therapeutic use. This acoustic technique uses a piezoelectric transducer to transform electrical energy into mechanical motion allowing the delivery of high‐energy pulses in a spatially coordinated manner with minimized undesired damage. Audible sound with a low frequency (20–20 000 Hz) did not directly interact with matter at molecular levels. However, audible sound‐induced liquid vibrations can be considered as periodic cycles of compaction and expansion of air, leading to a periodic increase and decrease of atmospheric pressure, respectively. This controls the dissolution of atmospheric gases (such as O_2_ and CO_2_) in solution to generate spatiotemporal chemical patterns. In addition, sound‐induced liquid vibration causes the solvent molecules to diffuse parallel to the walls of the container. The temporal control of mechanical forces exerting on supramolecular systems is facilely achieved by adjusting the duration time of force application. The spatial selectivity of mechanical stimuli is relatively weak since these external forces are normally applied to bulk materials or solution. Techniques like focused ultrasound and located compression are able to spatially control the mechanical forces.

## 3 Mechano‐responsive Covalent Bonds and Noncovalent Interactions

### 3.1 Breakage of Covalent Bonds

**Figure 2:**
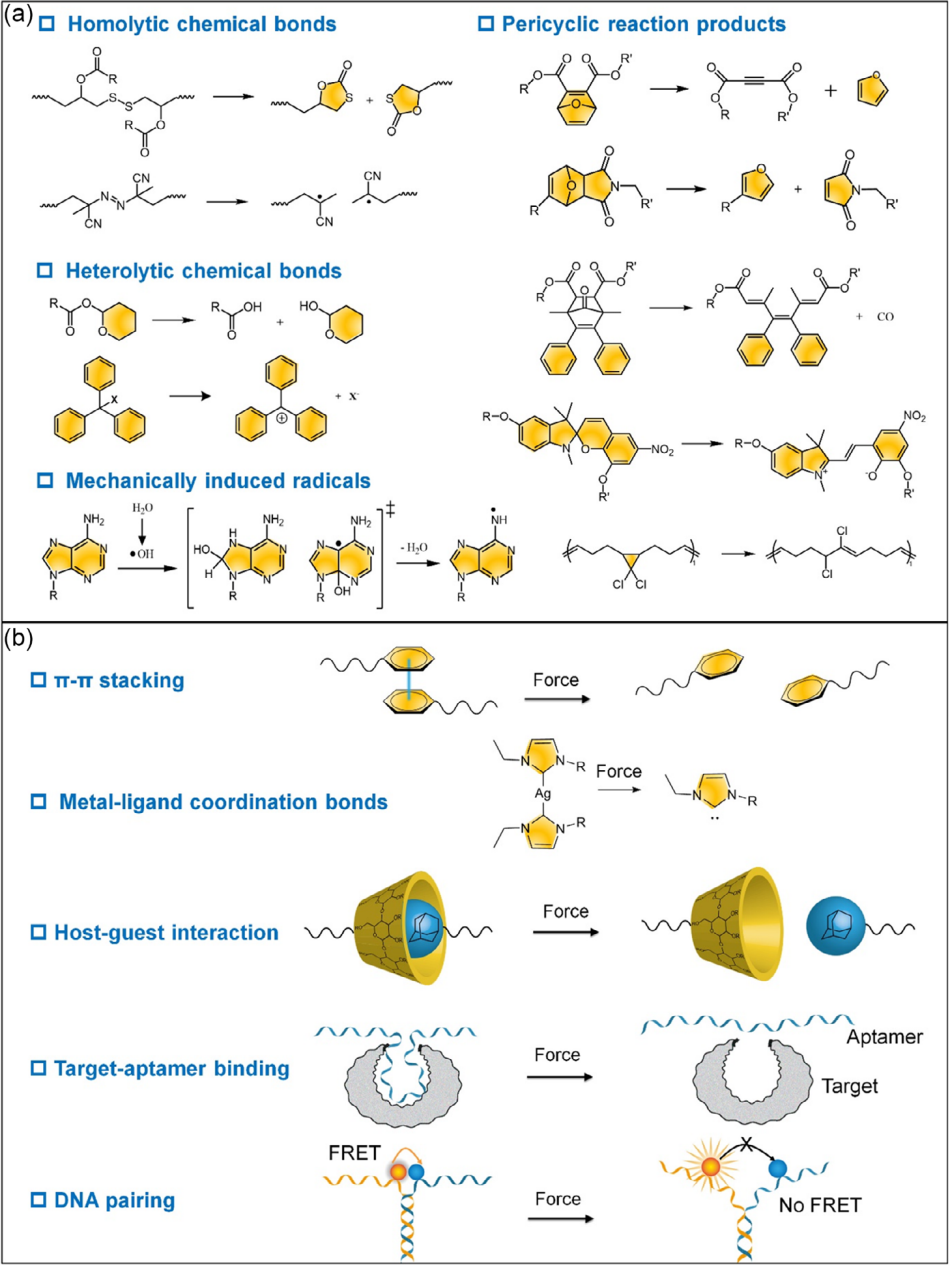
Mechanically responsive chemical and physical interactions. a) Mechanically active chemical bonds are categorized as heterolytic chemical bonds, hemolytic chemical bonds, pericyclic reaction products, and organic groups responsive to mechanically induced radical. b) Mechanical stimuli modulates non‐covalent interactions in supramolecular systems consisting of π–π stacking, coordination bonds, host–guest interaction, target‐aptamer recognition, and DNA hybridization.

Materials exposed to significant external stress typically undergo mechanochemical reactions, often leading to the indiscriminate breaking of covalent bonds. This results in potential damage or even failure of the material. However, a strategic alternative exists: the deliberate molecular design of synthetic materials that incorporate mechanically sensitive chemical groups, termed “mechanophores” (**Figure** **2**). By doing so, mechanical stress can be channeled to beneficially modify material properties.[^13^, ^15^] Mechanophores, based on their reactive mechanisms, can be categorized into five main groups: homolytic breakage, heterolytic breakage, pericyclic reactions, and radical‐induced bond breakage. Homolytic bond cleavage occurs to weak chemical bonds such as peroxide,^[^ ^16^ ^]^ bis(9‐methylphenyl‐9‐fluorenyl) peroxide,^[^ ^17^ ^]^ diazo^[^ ^18^ ^]^ and disulfide^[^ ^19^ ^]^ within a mechanically stressed environment, which stretch and break the covalent bonds homolytically. Heterolytic bond cleavage occurs to mechanophores such as triarylmethane,^[^ ^20^ ^]^ N‐heterocyclic carbene,^[^ ^21^ ^]^ and cyclic poly(*o*‐phthalaldehyde)^[^ ^22^ ^]^ that are known to undergo heterolytic bond cleavage when subject to mechanical forces, resulting in an unequal distribution of two unbound fragments with opposite charges. Pericyclic reactions can be mechanically induced when mechanophores are incorporated as latent force‐responsive groups within polymer architectures. One established approach to reduce the internal stability of molecules is the formation of strained cyclic structures.^[^ ^23^ ^]^ The mechanophores that undergo pericyclic reactions include cyclobutane,^[^ ^24^ ^]^ *β*‐lactam,^[^ ^24^ ^]^ spiropyran,^[^ ^12^, ^25^ ^]^ rhodamine,^[^ ^26^ ^]^ *gem*‐dihalocyclopropane,^[^ ^27^ ^]^ alkynes‐furan, and furan‐maleimide adducts.^[^ ^28^ ^]^ In addition, the ultrasound stimulation of free radicals in water introduces a novel mechanism for designing indirectly responsive molecules to mechanical forces. The application of ultrasound generates “cavities”, creating localized areas of high temperature and pressure, along with the collapse of bubbles. During sonication, water molecules can produce atomic hydrogen, hydroxyl radicals, and hydrated electrons (e^−^).^[^ ^29^ ^]^ The advancement of various mechanophores has paved the way for innovative applications including the activation of latent catalysts,^[^ ^30^ ^]^ targeted molecule release,[^19^, ^28^] the development of self‐strengthening materials,^[^ ^31^ ^]^ and the synthesis of semiconducting polyacetylene.^[^ ^32^ ^]^

### 3.2 Disruption of Non‐covalent Interactions

Beyond covalent bonds, supramolecular systems harness the power of multiple non‐covalent interactions to facilitate the self‐assembly of building blocks, granting them the ability to respond to mechanical forces (Figure 2b). Among these interactions, hydrogen bonds play a pivotal role in various biological processes, including DNA hybridization and protein folding. On average, the strength of hydrogen bonds is considerably lower than that of covalent bonds. In aqueous solutions, hydrogen bonds exhibit high dynamics, continually breaking and reforming. Supramolecular systems, such as DNA structures, held together by multiple hydrogen bonds, can react to external mechanical forces like sonication and stretching.^[^ ^33^ ^]^ Directional metal‐ligand coordination bonds can be readily disrupted by mechanical forces, such as ultrasonic cavitation. For instance, coordination polymers, such as Pd‐phosphane telechelic polytetrahydrofuran, Cu‐terpyridine, and Ag‐NHC, have been reported to be mechanically cleavable in homogeneous solutions, which reduced the molecular weight of polymers.^[^ ^34^ ^]^ The disruption of these bonds was facilitated by the collapse of cavitation bubbles generated by ultrasound, which imparted shear forces to induce breakage in the polymer chains. This approach is also applied to mechanically activate latent catalysts through mechanochemical bond scission.^[30a]^ Furthermore, neutral metallocenes like ferrocene and ruthenocene, as well as cationic cobaltocenium, have been observed to selectively respond to mechanical scission through various mechanisms of mechanophore dissociation.^[^ ^35^ ^]^

Another essential non‐covalent interaction is π–π interaction, which occurs between aromatic rings. This interaction is vital in driving the self‐assembly of conjugated molecules like oligo *p*‐phenylenevinylene and perylene diimides. Notably, it also plays a crucial role in maintaining the thermal stability of proteins and DNA helices. The hydrophobic effect stands out as a dominant force responsible for desolvation and the creation of lipid bilayers.^[^ ^36^ ^]^ This phenomenon governs the structural organization of substances like soaps, micelles, biomolecules, and other amphiphilic or nonpolar systems in water. In the hydrophobic effect, the preference of hydrophobic solutes in an aqueous solution to maximize water–water interactions leads to the aggregation of hydrophobic groups in the solution. Furthermore, host–guest complexation allows for the partial or complete encapsulation of guest molecules within the cavities of macrocyclic host molecules, such as cyclodextrin. These relatively weak, dynamically changing bonds are in constant equilibrium and are highly susceptible to external conditions, including mechanical forces.

## 4 Mechanically Responsive Supramolecular Systems

### 4.1 Supramolecular Assembly of π‐Conjugated Molecules

**Figure 3:**
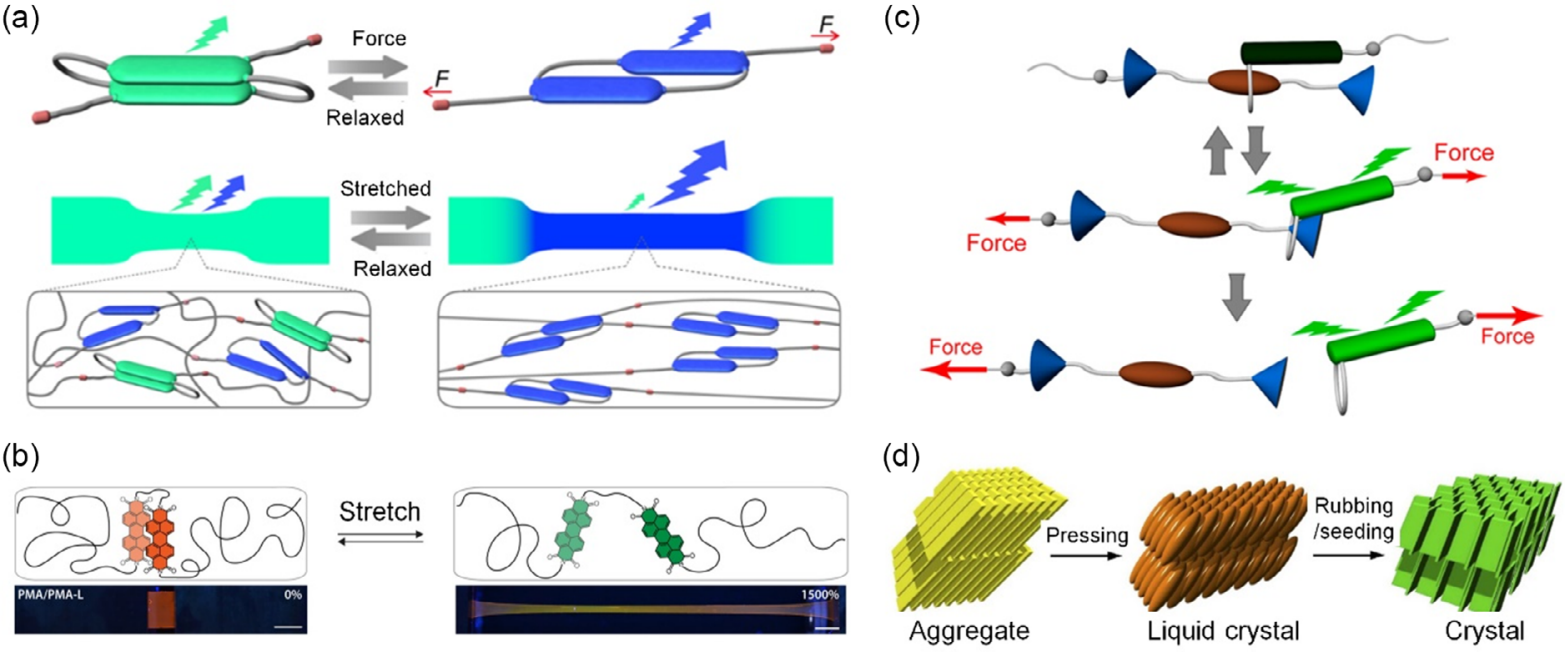
Force‐controlled π‐conjugated supramolecular systems. a) Cyclophane‐based supramolecular mechanophore and the mechanochromic luminescence of a mechanophore‐containing polymer. b) Poly(methyl acrylate)s containing a perylene diimide loop in their backbone. Mechanical deformation disrupts intramolecular interactions among the dyes and thereby changes the emission color from orange (excimer) to green (monomer). c) A rotaxane‐based supramolecular mechanophore that displays mechanical responses. In the force‐free state, the cycle carrying the fluorophore (dark green) is located in the proximity of a quencher (brown), and the fluorescence is quenched. The application of force separates the quencher from fluorophore to switch on its fluorescence. This effect is reversible unless the force is too high. The motif is equipped with two reactive groups (gray) that permit the integration into polymer chains. d) Schematic illustration of mechanically responsive phase transition of amphiphilic dipolar π‐systems. a) Adapted with permission.^[^ ^39^ ^]^ Copyright 2021, American Chemical Society. b) Adapted with permission.^[^ ^40^ ^]^ Copyright 2021, Wiley‐VCH. c) Adapted with permission.^[^ ^41^ ^]^ Copyright 2021, American Chemical Society. d) Adapted with permission.^[^ ^42^ ^]^ Copyright 2014, Springer Nature.

Supramolecular assemblies that incorporate host–guest pairs or π‐stacking fluorophores can exhibit responsive fluorescence or luminescence when subjected to external mechanical forces.^[^ ^37^ ^]^ For instance, Weder and Pucci^[^ ^38^ ^]^ embed the π‐stacking molecules into thermoplastic elastics to activate the fluorescence response to deformation of elastics. Furthermore, Sagara and Weder^[^ ^39^ ^]^ reported a mechanically responsive cyclic compound containing two fluorescent 1,6‐bis(phenylethynyl)pyrene groups that formed intramolecular excimers (**Figure** **3**a). This mechanophore was covalently integrated into polyurethane elastomer films, displaying a substantial excimer emission. Upon deformation (strain exceeding 600%), the fluorescence shifted toward a monomer‐dominated state, accompanied by a change from cyan to blue in color. This strategy has also been applied to create mechanoresponsive materials by incorporating folded perylene diimide (PDI) loops within polymer networks. In this design, two PDIs were linked by a short spacer and incorporated into poly(methyl acrylate) networks using atom transfer polymerization (Figure 3b). In the relaxed state, the motif formed a loop excimer. However, upon mechanical deformation (200% and 600% of strain), the PDI loops underwent conformational unfolding, resulting in a visibly discernible change in fluorescence color.^[^ ^40^ ^]^ In addition, mechanical force was employed to reversibly and irreversibly control rotaxane‐based supramolecular mechanophores through force‐induced dethreading of rotaxanes (Figure 3c).^[^ ^41^ ^]^ These rotaxane mechanophores consisting of a fluorescent host ring threaded onto an axle with a quencher. In the absence of stress, the fluorophore was positioned close to the quencher, leading to fluorescence quenching. Application of stretching (strain exceeding 600%) caused the fluorophore to move away from the quencher, resulting in the activation of fluorescence. In these systems, fluorescent π‐conjugated mechanophores are covalently incorporated into the polymer chains and assemble to form stacked aggregates or host–guest complex. Polymer entanglement results in the formation of networking elastic materials. The presence of mechanical tension causes fluorophores dislocation and weakens the π–π stacking and host–guest interactions, which results in the changes in fluorescence emission. Although the energy to break non‐covalent bonds is low, this strategy is not very efficient since most of the mechanical energy applied is preferentially used to stretch the polymer chains. As such, fluorescence response is activated only when a large strain is applied (200%–600%). In an alternative approach, compression or gentle grinding were utilized to induce transitions in the metastable self‐assembly states of amphiphilic dipolar‐conjugated systems, leading to stable packing arrangements and significant changes in luminescence due to distinct intermolecular packing (Figure 3d).^[^ ^42^ ^]^

### 4.2 Mechanically Triggered Gelation

**Figure 4:**
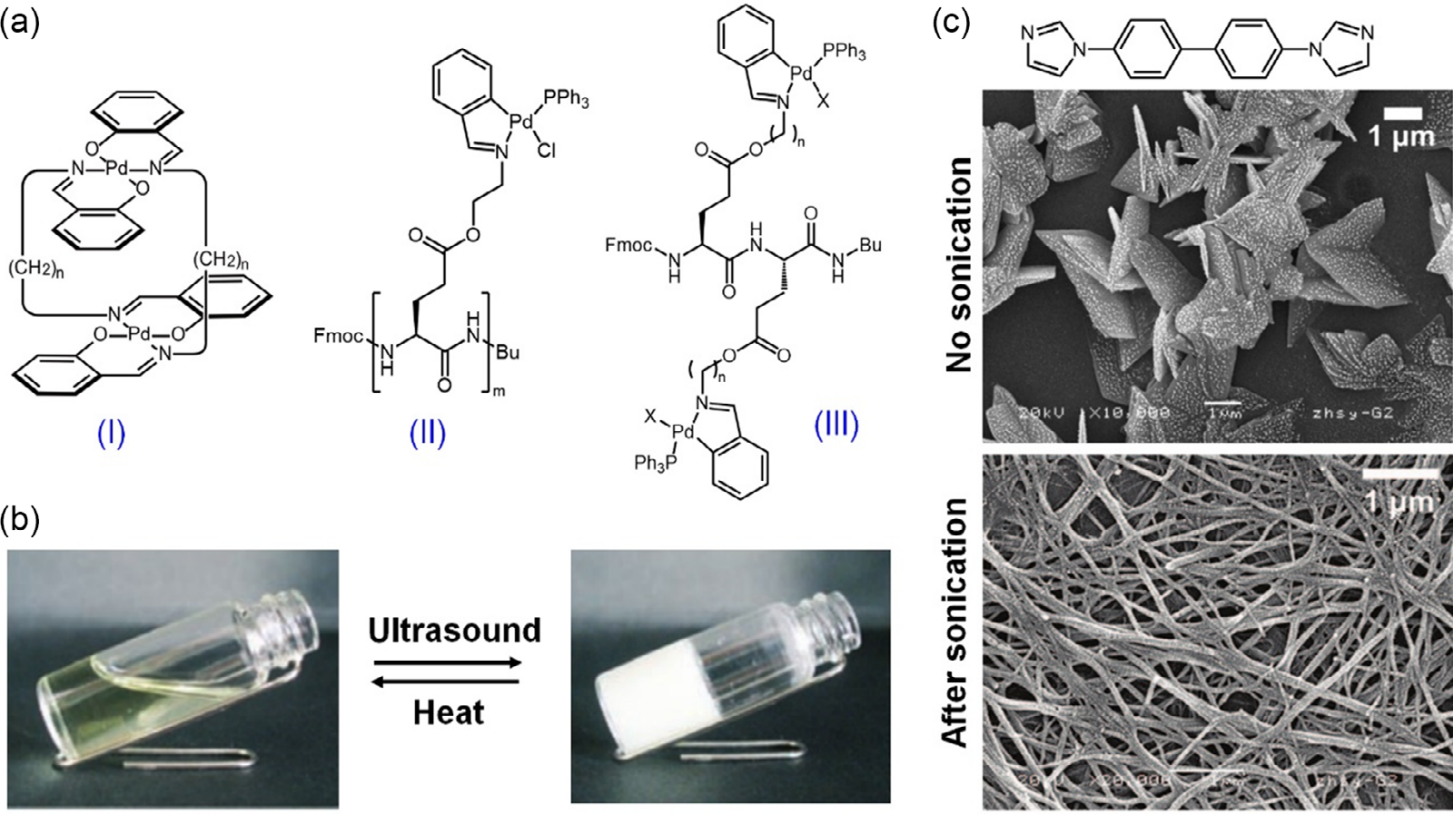
Sonication‐triggered supramolecular gelation. a) Molecular structures of metallo‐amphiphiles that respond to ultrasound. b) Photos showing ultrasound‐triggered transition from a transparent pale‐yellow solution to an opaque gel. c) An imidazole‐derived ligand and ultrasound‐induced morphological transition from sheet‐like structures to elongated fibrils, which is accounted for organogelation. a) Adapted with permission.^[^ ^44^ ^]^ Copyright 2005, American Chemical Society. b) Adapted with permission.^[33a]^ Copyright 2007, Wiley‐VCH. c) Adapted with permission.^[^ ^76^ ^]^ Copyright, American Chemical Society.

Mechanical forces like ultrasound can intensify the translational motion of molecules or particles in a solution, which might be expected to disrupt the weak noncovalent interactions involved in self‐assembly.^[^ ^43^ ^]^ However, carefully designed supramolecular systems can undergo gelation induced by sonication through alterations in molecular conformation, shifts in the self‐assembly pathway, or the oxidation of amphiphiles. For instance, Naota and coworkers^[^ ^44^ ^]^ synthesized a dinuclear Pd complex that was stabilized by π‐stacking interactions (**Figure** **4**a, compound I). Initially, the aggregation of this complex was hindered by its clothespin‐like, bent conformation. Exposure to ultrasound (40 kHz, 0.45 W cm^−2^, 0–15 s) prompted a conversion from self‐locking to interlocking configurations, resulting in the formation of a small amount of heterochiral, interpenetrative dimers. This, in turn, induced polymerization and gelation. Similarly, ultrasound‐induced gelation was observed in hydrogen‐bonded aggregates within a newly designed metalated dipeptide with switchable self‐lock/interlock capabilities (Figure 4a, II and III). Ultrasound irradiation (40.0 kHz, 0.45 W cm^−2^, 60 s) released the self‐lock of palladium‐bound peptides, leading to the formation of semi‐stable initial aggregates and initiating β‐sheet aggregation (Figure 4b).^[33a]^ Sonication (40.0 kHz, power 0.28 W cm^−2^) of dipeptide dispersions in alkanes resulted in homogeneous liquid gelation, where large sheets transformed into an extended network of thinner fibers, contributing to gel formation.^[^ ^45^ ^]^ Moreover, sonication was found to alter the self‐assembly pathway of coordination polymers, reshaping sheet‐like particles into elongated structures, which played a role in the formation of supramolecular gels (Figure 4c).^[^ ^43^, ^45^ ^]^ In these systems, the process of gelation was triggered by sonication, which modified the molecular conformation or the self‐assembly pathway. Yet, it remains uncertain whether these supramolecular gels reach thermodynamic equilibrium or maintain long‐term stability. Additionally, the predominantly destructive nature of sonication on soft materials casts doubts on the method's universal applicability.

An alternative approach involves using liposomes to encapsulate hydrogelation promoters or activators that can be mechanically released. For example, Stevens and coworkers^[^ ^46^ ^]^ employed ultrasound‐triggered enzyme catalysis to induce hydrogelation. In their design, ultrasound (20 kHz, 20% amplitude and 25% duty cycle) ruptured liposomes, releasing calcium ions, which served as activators for transglutaminase. This enzyme catalyzed intermolecular covalent crosslinking between lysine and glutamine sidechain residues of fibrinogen, resulting in fibrinogen hydrogel formation. In a liposome‐embedded polymeric hydrogel system, Pang and Liu^[^ ^47^ ^]^ demonstrated that hydrogel swelling could deform liposomes, triggering content release. This content release, in turn, further crosslinked the polymer networks, reinforcing their mechanical strength.

### 4.3 Chiral Self‐Assembly

**Figure 5:**
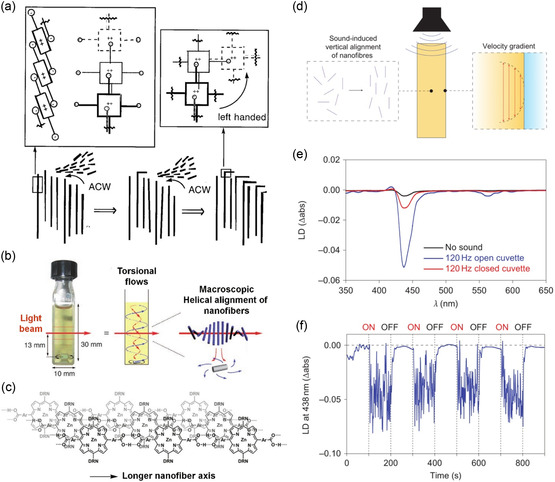
Mechanically responsive supramolecular chirality. a) Schematic illustration of chiral self‐assembly of J‐aggregated porphyrin. Diasterotropic relative motion of oligomeric blocks and steric hindrance effects the folding of J‐aggregates, which eventually results in sign induction by vortical stirring. Insets: 3D extended chromophores in J‐aggregates. b) Rotary stirring of a solution in a quartz cell with a magnetic stirring bar results in torsional flows and helical alignment of self‐assembled nanofibers. c) Self‐assembly of J‐aggregated zinc porphyrin dendrimers into hydrogen‐bonded nanofibers. d) Vertical alignment of nanofibres induced by sound vibration (left) and the resulting velocity gradient between the centre and sidewalls of the cuvette (right). e) LD spectra of the solution with (blue curve) and without (black curve) sound irradiation, and contained in a closed cuvette with sound irradiation (red curve). f) Repeated ON‐OFF switch of LD in response to sound irradiation. a) Adapted with permission.^[^ ^49^ ^]^ Copyright 2001, AAAS. b,c) Adapted with permission.^[^ ^51^ ^]^ Copyright 2007, Wiley‐VCH. d–f) Adapted with permission.^[^ ^53^ ^]^ Copyright 2010, Springer Nature.

In the realm of supramolecular chemistry, symmetry breaking is typically induced and amplified by chirality and aggregation. However, an intriguing discovery has revealed that mechanical force can induce symmetry breaking in supramolecular systems, particularly under non‐equilibrated conditions. The investigation of such spontaneous emergence of chiroptical activity from achiral building blocks represents a significant stride toward understanding the origin of chirality in the natural world.^[^ ^48^ ^]^ Ribó et al.^[^ ^49^ ^]^ reported that the direction of vortex stirring could induce chirality in a supramolecular structure dissolved in a solution, resulting in biased symmetry breaking (**Figure** **5**a). In this study, the electrostatic J‐aggregates of achiral diprotonated meso‐sulfonatophenyl‐substituted porphyrins became optically active when subjected to rotary stirring. This transformation was interpreted in terms of hydrodynamic and steric effects during the growth of supramolecular homoassociates. Aida and his colleagues^[^ ^50^ ^]^ demonstrated that self‐assembled J‐aggregates of dendritic zinc porphyrin could form a chiroptically active film using a spin‐coating process (6000 rpm, Figure 5b,c). Interestingly, the direction of spinning selected the chiral sign of the resulting film. Subsequently, J‐aggregates of zinc porphyrin dendrimers were used to spectroscopically visualize vortex flows. Rotary stirring at 1350 rpm induced temporary helical alignment of long nanofibers at the macroscopic level, allowing for the chiroptical visualization of torsional flows within the generated vortex.^[^ ^51^ ^]^ It is worth noting that the chiroptical activity in this context did not stem from the helical twisting of the supramolecular nanofibers of porphyrin derivatives within the vortex flow. Meijer and coworkers^[^ ^52^ ^]^ reported that the circular dichroism response of a dilute solution of an achiral oligo(*p*‐phenylene vinylene) derivative became stronger after subjecting the solution to mechanical shaking. While it is known that ultrasonic vibrations with frequencies exceeding 1 MHz can align specific macromolecules in solution, research on the impact of audible sound with much lower frequencies (20–20 000 Hz) is relatively rare. Aida and his team further contributed to this area by designing a supramolecular nanofiber that exhibited preferential alignment parallel to the direction of audible sound propagation (120 Hz, Figure 5d–f).^[^ ^53^ ^]^

### 4.4 Out‐of‐Equilibrium Systems

**Figure 6:**
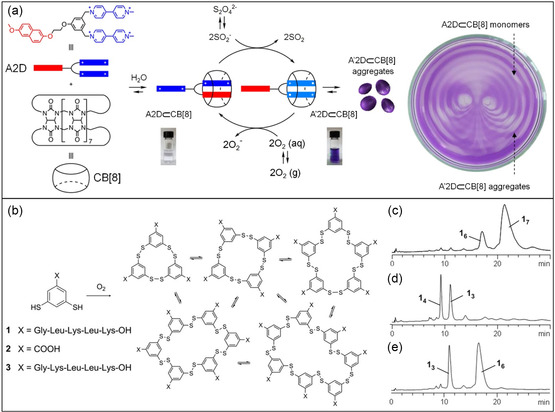
Mechanically active non‐equilibrium systems. a) Sound‐controlled generation of spatiotemporal pattern and the corresponding reversible chemical transformations in an out‐of‐equilibrium cycle. Transient aggregation of a host–guest complex between CB[8] and a viologen derivative is governed by reduction and oxidation. The reduced state A′2D⊂CB[8] shows the aggregation of supramolecular complex. b) A dynamic combinatorial peptide library. c–e) High performance liquid chromatography profiles of cyclic peptide products obtained by oxidation of peptide **1**: c) stir for 15 days (1200 rpm); d) no agitation after 16 days; and e) shake for 20 days (500 rpm). a) Adapted with permission.^[^ ^55^ ^]^ Copyright 2020, Spring Nature. b–e) Adapted with permission.^[^ ^57^ ^]^ Copyright 2010, AAAS.

Living organisms maintain a state far from equilibrium by harnessing biological and chemical fuels to support complex biological functions. Efforts have been directed toward designing synthetic systems that emulate the non‐equilibrium phenomena found in nature. Synthetic out‐of‐equilibrium chemical oscillating networks, like the Belousov–Zhabotinsky reaction, have been devised to control spatiotemporal cycles.^[^ ^54^ ^]^ Kim and his collaborators^[^ ^55^ ^]^ employed sound waves as a guiding stimulus to generate spatiotemporal patterns in out‐of‐equilibrium chemical reactions and self‐assembly systems (**Figure** **6**a). The application of audible sound (40 Hz, threshold acoustic intensity of 0.06 W m^−2^ and a pressure 5.0–6.3 Pa) induced liquid vibrations controlled the dissolution of atmospheric gases (such as O_2_ and CO_2_) in water, resulting in the creation of spatiotemporal chemical patterns within the fluid. This segregation produced solution domains with differing redox properties or pH values, enabling patterned supramolecular complexes and self‐assembly. Recently, Zhao and coworkers^[^ ^56^ ^]^ demonstrated how shaking can control non‐equilibrium supramolecular polymerizations in a closed system composed of alkyl‐substituted viologens and pyranine. Co‐assembly of pyranine and alkyl‐substituted viologen led to the formation of micrometer‐sized nanotubes. The presence of hydrazine hydrate reduced the viologens and disassembled nanotubes over time, which were subsequently oxidized by air during shaking. This mechanosensitive dissipative system was also extended to create a chiral transient supramolecular helix and produce template‐free reproducible patterns. In both cases, mechanical stimuli from ultrasound or shaking affect the diffusion rate of oxygen in a specific direction within the solution, resulting in unique visible patterns arising from mechano‐sensitive redox reactions.

Otto and coworkers^[^ ^57^ ^]^ demonstrated the significant role of mechanical forces, namely shaking (500 rpm) and stirring (1200 rpm), in governing the self‐replication of peptide macrocycles originating from a dynamic combinatorial library (Figure 6b). The oxidation of peptides led to the formation of disulfide bonds and a range of macrocycles with varying tendencies to aggregate into fibrils. In the absence of agitation, trimer, and tetramer structures prevailed. However, when subjected to stirring and shaking, the proportion of cyclic hexamers and heptamers increased. This mechano‐sensitivity resulted from the breakage of fibrils, creating more active ends that facilitated fibril replication. These findings illustrate how mechanical forces can serve as a selective pressure in the competition between replicators, ultimately determining the outcome of covalent synthesis.

### 4.5 Deformation, Division, and Polarity Switch of Vesicles

The preparation of phospholipid vesicles typically involves the input of mechanical energy, such as sonication and extrusion, to balance the bending energy. Mechanical forces, including shear and ultrasound, applied to vesicular compartments play a crucial role, particularly in drug delivery application.^[^ ^58^ ^]^ These mechanical forces are harnessed to induce shape deformation, division, ion transportation, and polarity switching in lipid vesicles.^[^ ^59^ ^]^ For instance, shear forces can induce a reversible shift in the melting temperature of giant unilamellar vesicle membranes. This is achieved by directly causing the formation of membrane pores with relatively long lifetimes in close proximity to the phase transition.^[59b,c]^ To introduce mechanical responsiveness, Bruns and coworkers^[59d]^ prepared self‐assembled polymersomes with amphiphilic block copolymers incorporating nucleobases in the hydrophobic block. The exposure to shear forces through a syringe needle (flow rate 70 mL min^−1^) caused the separation of nucleobase pairs in the hydrophobic leaflet, exposing their hydrogen bonding motifs, which transiently switched the vesicular membrane to semipermeable states. The permeability switch was utilized for on‐demand payload release from the polymersomes and to activate biocatalytic reactions within their interior.^[59d]^ Dekker and coworkers^[59e]^ introduced a microfluidics‐based method to drive the mechanical division of cell‐sized liposomes. When encountering a Y‐shaped bifurcation in a microfluidic channel at high flow velocity (510 mm s^−1^), the liposomes undergo deformation and, remarkably, divide into two stable daughter liposomes in just a few milliseconds. The probability of successful division depended critically on the surface area‐to‐volume ratio of the mother liposome, which could be adjusted through osmotic pressure. It also strongly correlated with the size of the mother liposome relative to the microchannel dimensions. Indeed, the field of mechanically sensitive vesicles has seen significant research focusing on various methods to induce membrane disruption for drug delivery applications. Two common methods are ultrasound‐induced sonoporation and shear‐induced deformation. While the steady‐state response of lipid membranes to mechanical forces is relatively well understood, real‐time measurement of membrane physics at the molecular level remains challenging. Techniques such as atomic force microscopy (AFM), optical tweezers, and microfluidic platforms have been employed to study membrane deformation and dynamics in real‐time. However, the mechanically triggered changes in membrane morphology, mechanical properties, and molecular interactions in a complicated and dynamic environment (e.g., bloodstream) is less understood.

## 5 Biomimetic Applications

### 5.1 Protein Activation

**Figure 7:**
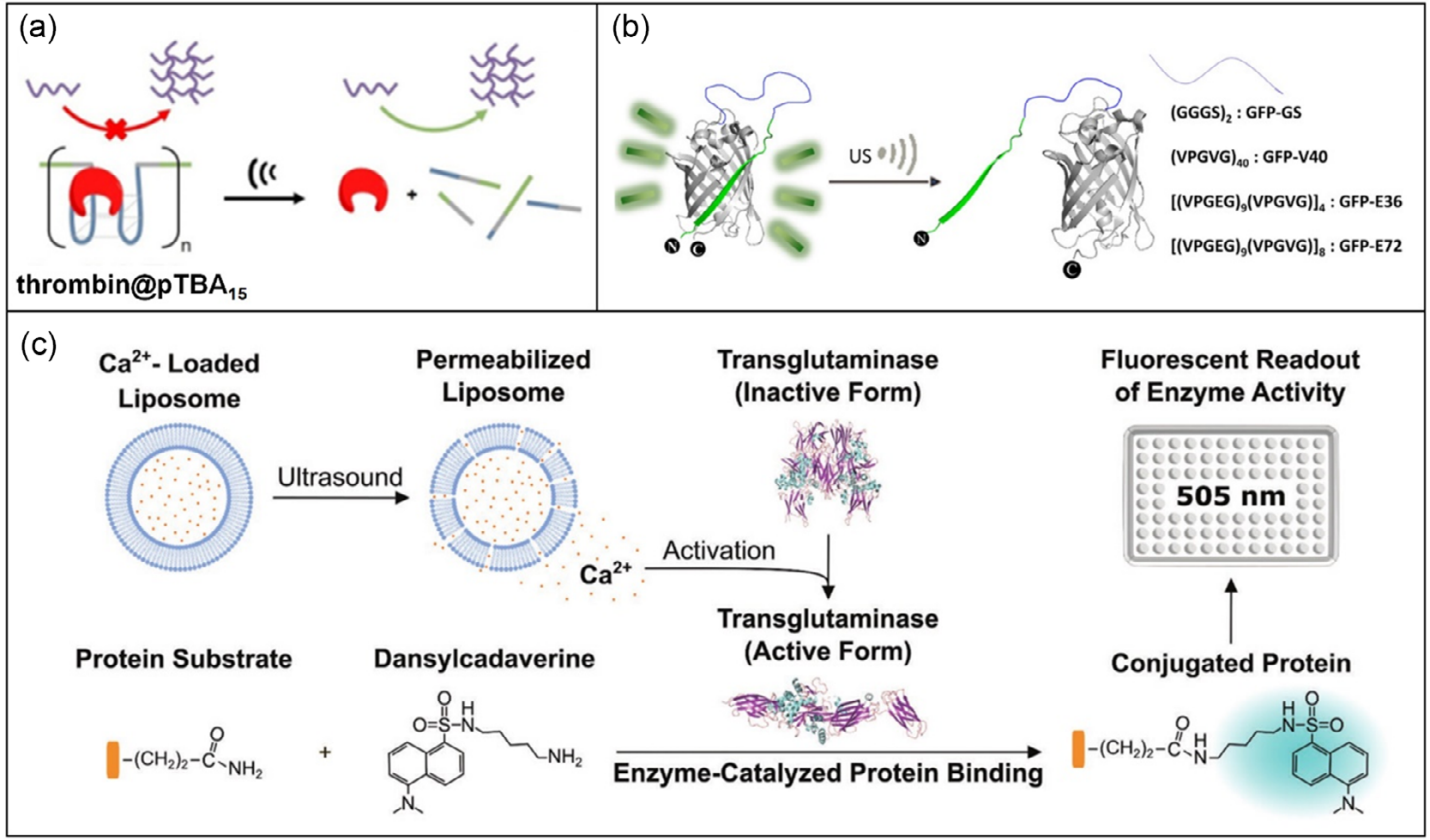
Ultrasound‐activated protein in supramolecular systems. a) The formation of thrombin‐aptamer complex (thrombin@pTBA_15_) inhibits enzyme activity, which is restored to catalyze the formation of fibrin from fibrinogen upon exposing to ultrasound. b) Ultrasound removes the binding of an anionic supercharged polypeptide from green fluorescence protein (GFP). c) Ultrasound‐triggered liposome destruction and the release of calcium ions, leading to the activation of transglutaminase. This enzyme catalyzes the conjugation of a substrate to protein, leading to the enhancement of substrate fluorescence. a) Adapted with permission.^[^ ^61^ ^]^ Copyright 2021, Wiley‐VCH. b) Adapted with permission.^[^ ^62^ ^]^ Copyright 2010, Wiley‐VCH. c) Adapted with permission.^[^ ^46^ ^]^ Copyright 2020, Wiley‐VCH.

Mechanical stimuli, particularly ultrasound irradiation, can be detrimental to proteins, causing unfolding, aggregation, nonspecific bond scission within the polypeptide backbone, and modifications of amino acid side groups.^[^ ^60^ ^]^ Recent research, however, has unveiled that moderate‐intensity ultrasound can activate proteins by disrupting the weak non‐covalent forces that bind proteins to DNA or polypeptides, or by releasing enzyme activators. For instance, Huo and Herman^[^ ^61^ ^]^ reported the ultrasound‐induced activation of thrombin, a key player in secondary hemostasis (**Figure** **7**a). Initially, polyaptamers were designed to specifically bind to thrombin, inhibiting its enzyme activity. The presence of ultrasound (20 kHz or 5 MHz, 30–300 s) generates inertial cavitation, disrupting the polyaptamer‐thrombin interaction and thereby activating the enzyme. Thrombin's activity can also be suppressed using split aptamers attached to the surface of gold nanoparticles. In this scenario, plasmonic gold nanoparticles aggregated, deactivating thrombin. Ultrasound application weakened the polyaptamer‐thrombin affinity, causing the disassembly of gold nanoparticle clusters and, consequently, activating the enzyme. Mechanical force (20 kHz, a power intensity of 7 W cm^−2^) could also destabilize the 11^th^ β‐strand of green fluorescent protein, resulting in the loss of fluorescence without altering the protein's secondary structure (Figure 7b). This principle has been applied to switch on enzyme activity.^[^ ^62^ ^]^ For this purpose, a long‐charged domain was fused to a peptide that inhibits trypsin. The application of ultrasound disrupted the interaction between the enzyme and inhibitor, ultimately releasing the active enzyme. More recently, Stevens and coworkers^[^ ^46^ ^]^ employed ultrasound (20 kHz, 20% amplitude, and 25% duty cycle) to release calcium ions from liposomes, triggering the catalysis of transglutaminase (Figure 7c). The ultrasound‐activated transglutaminase catalyzed intermolecular covalent crosslinking between lysine and glutamine sidechain residues of soluble fibrinogen molecules, resulting in the production of fibrinogen hydrogels.

### 5.2 Mechanically Triggered Drug Release and Therapeutics

The concept of mechanically triggered drug release holds significant appeal, primarily because these structures are well‐suited for encapsulating both hydrophilic and hydrophobic drugs.^[^ ^63^ ^]^ Mechanical stimuli, including compressive force, tensile force, shear, and ultrasound, have proven effective in activating drug release mechanisms. One promising approach involved cavitation‐facilitated microbubble‐mediated focused ultrasound therapy. This method offered several advantages, including non‐invasiveness, absence of ionizing radiation, substantial tissue penetration depth, and precise spatiotemporal control.^[^ ^64^ ^]^ Furthermore, ultrasound‐triggered drug release has been explored within various systems such as liposomes, micelles, microbubbles, and hydrogels.^[^ ^65^ ^]^ In this context, we will highlight some recently developed supramolecular systems that demonstrate controlled drug release and efficient therapeutic potential under mechanical fields.

**Figure 8:**
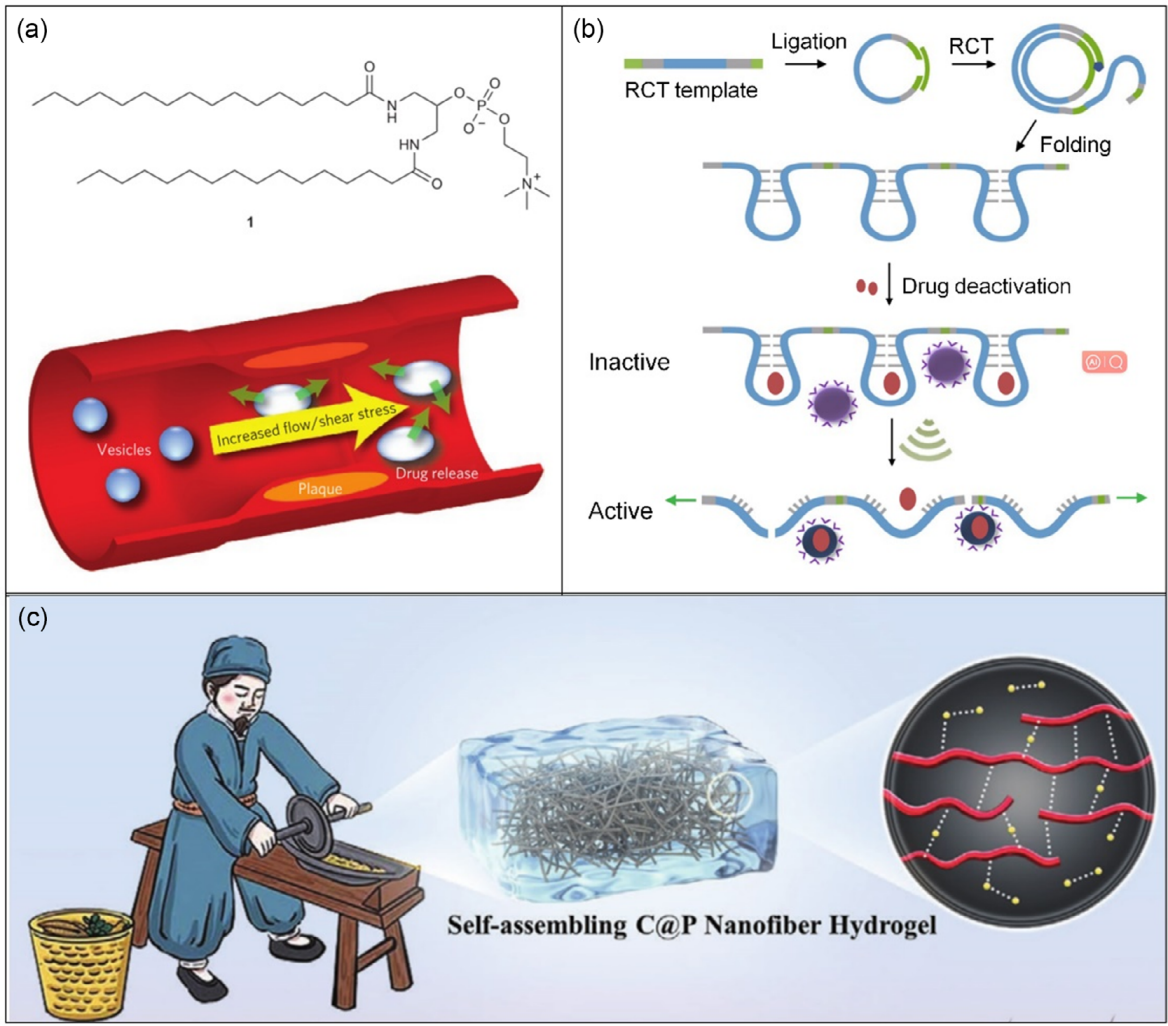
Mechanical activation of therapeutics. a) Molecular structure of 1,3‐diaminophospholipid and self‐assembled liposomes that undergo shape deformation in shear flow. b) Polyaptamers designed from a circular template are loaded with neomycin B, which are released by ultrasound‐induced stretching and bond scission. c) Mechanical grinding promotes the molecular collision and accelerate self‐assembly into therapeutic hydrogels. a) Adapted with permission.^[^ ^66^ ^]^ Copyright 2012, Springer Nature. b) Adapted with permission.^[19a]^ Copyright 2021, Springer Nature. c) Adapted with permission.^[^ ^69^ ^]^ Copyright 2022, Wiley‐VCH.

Narrowed arterial blood vessels in atherosclerosis lead to increased endogenous shear stress within constricted arteries. This property has been ingeniously harnessed to design mechanically responsive drug release systems from soft materials. For instance, Zumbuehl and his research team^[^ ^66^ ^]^ have developed mechanically sensitive liposomes using a 1,3‐diaminophospholipid (**Figure** **8**a). These liposomes remained stable under static conditions but released their contents upon exposure to vortex (2500 rpm, 60 s). This behavior can be attributed to their lenticular morphology, which led to instabilities along their equator. Using a model cardiovascular system, they demonstrated that drug release from these vesicles within constricted vessels was enhanced under higher shear stress conditions.

Ultrasound technology is another valuable tool for generating longitudinal pressure and inducing mechanical forces. Herrmann and colleagues[^19^, ^67^] have described three strategies for applying ultrasound to activate drug release from polymers or nano‐assemblies through the cleavage of both covalent and non‐covalent bonds (Figure 8b). In the first strategy, pulsed ultrasound (20 kHz, sonication time of 9600 s, sonication energy of 202292 J, sonication power of 21.07 W) cleaved a disulfide motif in a polymer to release an alkaloid drug from a β‐carbonate linker. In the second approach, multiple copies of RNA aptamers were synthesized using rolling circle transcription based on a template encoding the RNA aptamer. Complexation between antibiotics and these repeated RNA aptamers allowed for efficient drug loading, subsequently activated by mechanochemical cleavage of the nucleic acid backbone with pulsed ultrasound (20 kHz, sonication time of 1800 s, sonication energy of 4,551 J, sonication power of 2.53 W). Lastly, nanoparticle‐polymer and nanoparticle‐nanoparticle assemblies were stabilized by hydrogen bonds between antibiotics, such as vancomycin, and their complementary peptide targets. Ultrasound‐induced scission of these hydrogen bonds resulted in the release of antibiotics. Similarly, pulsed ultrasound (1.0 s on, 1.0 s off at 50% Amplitude) was demonstrated to selectively activate doxorubicin (DOX) loaded in DNA‐modified gold assemblies, where gold nanoparticles transmit shear force and DNA strands served as mechanically sensitive components.^[^ ^68^ ^]^ Ultrasound (1 MHz, 1.5 W cm^−2^ for 3 h) can also generate hydroxyl radicals and release Fe^2+^ from ferrocene, enhancing ferroptosis and reactive oxygen species (ROS) therapy in polymeric micellar systems.^[29a]^ Taking inspiration from the grinding treatment of traditional Chinese medicine, mechanical grinding has been introduced to promote effective molecular collision and accelerate the self‐assembly of chitosan and puerarin, leading to the fabrication of Chinese‐herb‐based hydrogels^[^ ^69^ ^]^ (Figure 8c).

### 5.3 Mechanical Sensors

**Figure 9:**
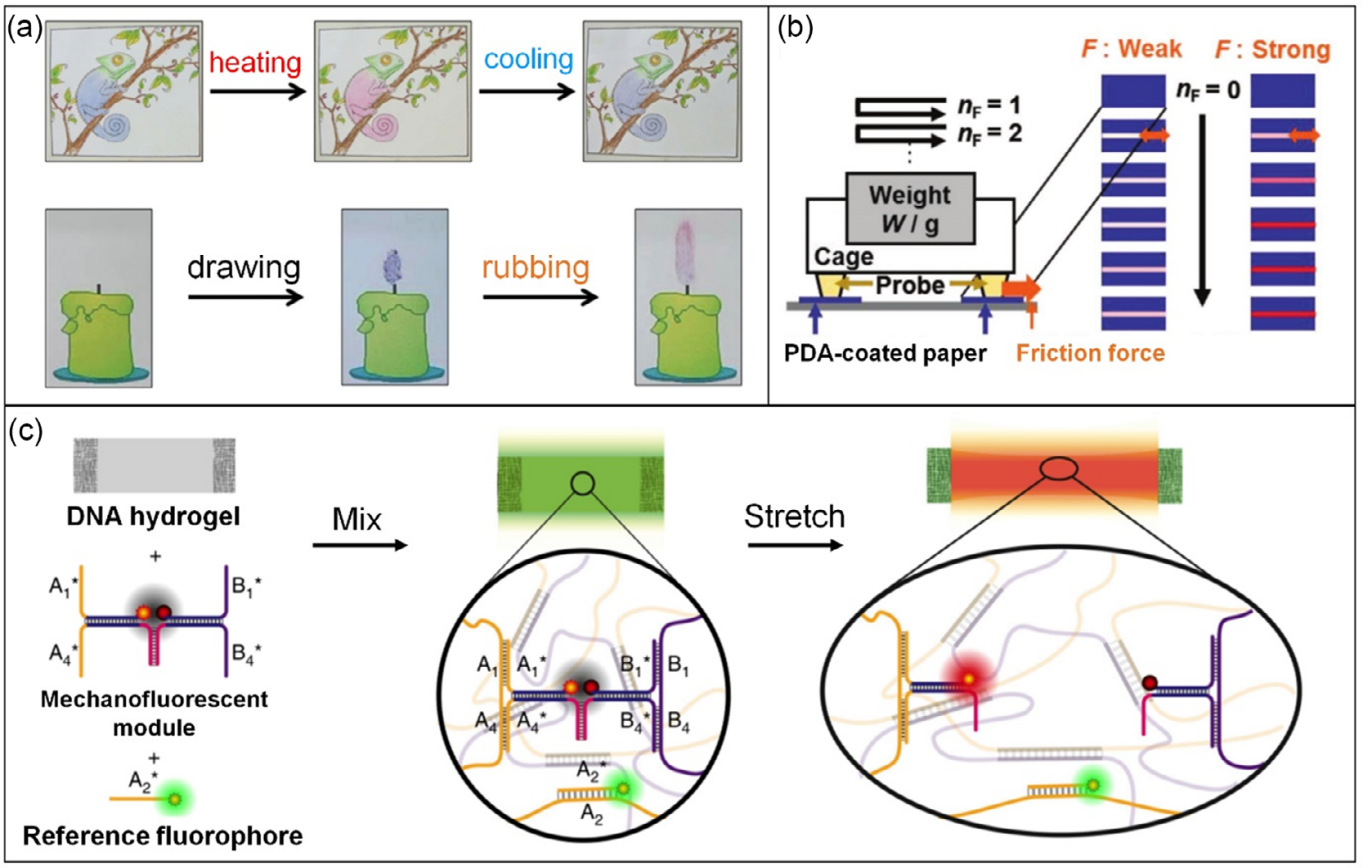
Design of mechanical sensors with supramolecular materials. a) A writable paper‐based conjugated polymer sensor derived from PDA‐wax composite. Top: photographs of a hand‐painted paper‐based sensor that experiences reversible colorimetric transition upon heating and cooling process. Bottom: photographs showing soft rubbing of the mechanically drawn image results in a colorimetric transition. b) Schematic illustration of the experimental setup for quantitative measurement of the applied friction force (F) on PDA‐coated paper sensors. c) Construction of DNA hydrogel functionalized with a FRET pair‐integrated force‐sensing module and a green‐emitting reference fluorophore. In the absence of stress, the hydrogel emits green fluorescence from reference fluorophore while red fluorescence is quenched in the force‐sensing module. Macroscopic stretching of the hydrogel breaks the DNA duplex of the mechanosensing module, resulting in restoration of red fluorescence by separating the red‐emitting fluorophore from the quencher. a) Adapted with permission.^[^ ^73^ ^]^ Copyright 2016, Wiley‐VCH. b) Adapted with permission.^[72e]^ Copyright 2018, Wiley‐VCH. c) Adapted with permission.^[33b]^ Copyright 2019, Springer Nature.

Mechanically responsive molecules or materials can be engineered to detect external stresses acting upon an object. A diverse array of mechanophores, such as spiropyran or rhodamine, has been incorporated into bulk materials to convert stress into colorimetric or fluorescent signals.^[^ ^70^ ^]^ However, activating mechanophores within bulk materials typically requires high levels of strain. The limited level of mechanochemical transduction is primarily attributed to the polymer network's topology.^[^ ^71^ ^]^ Alternatively, thoughtfully designed supramolecular systems have shown the capability to sensitively detect external mechanical stimuli. One example is conjugated polydiacetylenes (PDAs), known for their appealing structural, spectral, and optical properties.^[^ ^72^ ^]^ PDAs are typically synthesized through light‐triggered 1,4‐addition of diacetylene monomers, resulting in a polymeric backbone featuring alternating carbon‐carbon double and triple bonds. Depending on their molecular structures, diacetylene‐containing monomers readily aggregate into self‐assembled nanostructures (such as vesicles and fibers). These nanostructures possess specific geometric parameters that facilitate topochemical polymerization. The presence of extensively delocalized π‐electron networks within the conjugated backbone and conformational restrictions along the main backbone give rise to optical properties characterized by an absorption peak at approximately 640 nm and an intense blue color. External stimuli, such as solvents, pH changes, heat, metal ions, nucleic acids, proteins, enzymes, and microorganisms, can disrupt the planarity of the polymer backbone, causing a blue shift in the absorption spectra. Consequently, PDA‐based materials have been developed into optical sensors. For instance, Kim and colleagues^[^ ^73^ ^]^ introduced a hand‐writable PDA sensor produced through a sequential mixing‐molding polymerization method using a PDA‐wax composite (**Figure** **9**a). Rubbing the handwritten image with a finger or applying heat induces a colorimetric transition in these paper‐based sensors. More recently, a PDA and dry liquid‐integrated paper has been developed for the visual detection of weak compression stresses (ranging from 10^0^–10^3^ Pa). In this article sensor, liquid droplets are coated with solid particles at the liquid–air interface to prevent liquid coalescence. The presence of weak compression stress releases the interior liquid, wetting the layered PDA and causing a color change (Figure 9b).^[72e]^

In another innovative approach, mechanically responsive supramolecular systems incorporating distance‐dependent FRET (Förster resonance energy transfer) pairs or π‐stacking fluorophores have been designed to sense external forces.^[^ ^39^, ^40^, ^41^, ^74^ ^]^ Walther and colleagues^[33b]^ engineered stretchable DNA hydrogels as mechanofluorescent soft materials by integrating FRET‐based DNA tension probes into structured DNA hydrogel networks (see Figure 9c). When macroscopic stretching occurs, the DNA hydrogel break the hybridized DNA bonds, separating the fluorophore from the quencher and restoring fluorescence emission. The programmability of DNA structures and the force probe provided temporal control over fluorescence recovery during stress relaxation, enabling reversible and irreversible strain sensing.

## 6 Summary and Outlook

Supramolecular systems, constructed through the self‐assembly of functional units, have garnered significant attention due to their unique properties and dynamic complexity. Despite recent advancements in stimuli‐responsive supramolecular materials, several challenges persist in the design of mechanically responsive self‐assemblies. These challenges mainly stem from the inherently destructive nature of mechanical forces on molecular assemblies.

In general, mechanical forces exert their influence on self‐assembly either chemically, via covalent bonds, or physically, through non‐covalent bonds.^[^ ^75^ ^]^ The first strategy involves incorporating mechanically vulnerable bonds or mechanophores into the building blocks. Fortunately, recent progress in mechanochemistry has provided a toolbox of newly designed mechanophores, employing various mechanisms such as hemolytic chemical bonds, heterolytic chemical bonds, pericyclic reactions, metal‐ligand coordination, and radical‐capturing functional groups (**Table** **1**). This rapidly evolving field has unlocked a multitude of functions, including payload release, catalyst activation, radical generation, mechanoluminescence, and mechanochromism. These mechanically liable bonds are characterized by weaker bond dissociation energies compared to regular covalent bonds. The second strategy revolves around manipulating non‐covalent interactions with mechanical forces (**Table** **2**). It is noteworthy that the dissociation energies of non‐covalent interactions, such as the hydrophobic effect, π‐π stacking, hydrogen bonding, biological interaction, and host‐guest recognition, are significantly lower than those of covalent bonds found in mechanophores. For instance, the forces required to carry out a range of supramolecular processes such as DNA unzipping and protein unfolding is estimated to be 10–200 pN, much lower than that required for mechanophore activation and covalent bond breakage (200 pN–10 nN). Consequently, supramolecular systems are highly susceptible to mechanical stimuli at a much lower energy input, causing changes beyond the molecular scale, such as the fragmentation of peptide fibrils, alignment of J‐aggregates, vesicle disruption, hydrogel formation/breakage, enzyme activation, chirality operation and fibril alignment. Unlike bulk polymer materials that can be subjected to stretching and compression, most supramolecular systems are investigated in a solution state, making them vulnerable to flow, shaking, stirring, ultrasound, or sound vibrations. Among these, solution‐based ultrasound stands out as the most effective method for activating mechanosensitive supramolecular systems. Ultrasound applies mechanical forces to disrupt both covalent and non‐covalent bonds through acoustic cavitation, resulting in a series of effects, including vesicle rupture, covalent bond cleavage, changes in molecular conformation, alterations in self‐assembly morphology, and the dissociation of biomolecular complexes (e.g., protein–aptamer interactions). The application of mechanical stimuli with low power also opens new avenues for the development of ultrasensitive mechanical sensors and wide application in biological environments.

**Table 1:** Representative systems involving mechanically active chemical bonds

Types	Motifs	Force types	Responses or applications	References
Homolysis	Disulfide bond	Ultrasound	Thiol generation	[19a]
	Peroxyl bond	Compression	Force‐induced emission	[17, 77]
	Diazo	Ultrasound	Radical generation	[18]
Heterolysis	*o*‐phthalaldehyde	Ultrasound	Depolymerization	[22]
	Epoxide	Ultrasound	Ring‐opening	[78]
	Active ester	Ultrasound	Drug delivery	[79]
Coordination bonds	Pd‐diphosphane	Ultrasound	Polymer chain breakage	[34b]
	Ag/Ru‐NHC	Ultrasound	Catalyst activation	[30, 80]
	Cu‐terpyridine	Ultrasound	Depolymerization	[34a]
	Zn‐imidazole	Ultrasound	Gelation	[76]
	Rh/Ir‐ligand	Ultrasound	Gelation	[43]
	Metallocene	Tension	Metal‐ligand dissociation	[35]
Pericyclic reaction products	Furan‐alkyne [4 + 2] product	Compression	Depolymerization	[28d]
	Furan‐maleimide [4 + 2] product	Ultrasound	Payload release	[28, 81]
	Maleimide−anthracene product	Tension	Mechanoluminescence	[82]
	Norborn‐2‐en‐7‐one	Ultrasound	Co release	[83]
	β‐lactam	Ultrasound	Cycloelimination	[24a]
	Spiropyran	Tension, ultrasound or grinding	Mechanochromism	[70, 84]
	Spirothiopyran	Tension	Mechanochromism	[85]
	Diarylbibenzofuranone	Kneading	Mechanochromism	[86]
	1,2‐dioxetane	Tension	Chemiluminescence	[24b]
	*Gem*‐dichlorocyclopropane	Ultrasound	Ring open	[87]
Generation of radicals	Adenine	Ultrasound	Gelation	[29b]
	Ferrocene	Ultrasound	Generation of ROS	[29a]

**Table 2:** Representative examples involving mechanically active physical interactions

Types	Motifs	Force type	Responses	References
Hydrogen bonding	DNA	Tension	FRET	[33b]
	Peptide	Ultrasound	Gelation	[33a]
π‐π interaction	Oligo *p*‐phenylenevinylene	Compression	Fluorescence	[42]
	Perylene diimide	Tension	Fluorescence	[40]
	Dinuclear Pd complex	Ultrasound	Gelation	[44]
	Quinacridone derivatives	Ultrasound	Gelation	[88]
Host‐guest recognition	Adamantane‐cyclodextrin	Compression	Patterning	[89]
	Rotaxane	Tension	Fret	[41]
	Thrombin‐aptamer	Ultrasound	Generation of fibrinogen	[61]
	Dox‐dna	Ultrasound	Release of dox	[68]
Hydrophobic interaction	Liposome	Shear	Drug release	[66]
	Ca^2+^ loaded liposomes	Ultrasound	Enzyme activation and gelation	[46]
	Diazido‐loaded liposomes	Swelling	Hydrogel reinforcement	[47]
	Polymer vesicles	Shear	Catalysis and gelation	[59d]

In summary, despite the potentially destructive nature of mechanical forces, the activation of non‐covalent bonds within supramolecular systems can yield valuable properties. Notably, mechanical activation offers precise spatiotemporal control and introduces non‐equilibrium dynamics to self‐assembly systems. However, challenges lie ahead in future studies. Unlike well‐established external stimuli like pH, light, and enzymes, where chemical or physical changes in supramolecular systems are well‐controlled, the transduction of mechanical energy in solution is less precise. For biomedical applications, there is a growing need to construct multifunctional systems rooted in supramolecular chemistry that seamlessly integrate mechanical sensitivity, dynamic properties, and bioactivity. To achieve this, the integration of mechanically active chemical (covalent) and physical (non‐covalent) bonds is highly desirable. We anticipate that such research will open new avenues for the design of integrative materials for drug delivery and wearable devices with therapeutic applications.

## Conflict of Interest

The authors declare no conflict of interest.
